# Supplementary material for: Prevalence and Risk of Sarcopenia in Patients with Chronic Pancreatitis: Systematic Review and Meta-Analysis
Source: Nutrients. 2025 Feb 28;17(5):870. doi: 10.3390/nu17050870 (PMC11902046; doi:10.3390/nu17050870)
Supplement: Supplementary file 1 [file nutrients-17-00870-s001.zip › File S2. Search strategy.docx]

| *PubMed* | ("Sarcopenia"[MeSH] OR sarcopenia OR muscle loss OR muscle wasting OR muscle atrophy OR skeletal muscle depletion OR cachexia OR frailty) AND ("Chronic Pancreatitis"[MeSH] OR chronic pancreatitis OR pancreatic exocrine insufficiency OR PEI OR pancreatic disease) AND ("Body Composition"[MeSH] OR body composition OR "Muscle, Skeletal"[MeSH] OR skeletal muscle OR "Sarcopenia"[MeSH] OR "Exercise"[MeSH] OR exercise OR handgrip strength OR grip strength OR gait speed OR appendicular lean mass OR ALM OR skeletal muscle index OR SMI OR DXA OR dual-energy X-ray absorptiometry OR bioelectrical impedance OR BIA OR CT OR MRI) |
| --- | --- |
| *Embase* | *('Sarcopenia' OR 'muscle loss' OR 'cachexia') AND ('Chronic Pancreatitis' OR 'pancreatic exocrine insufficiency' OR 'PEI')* |
| *Google Scholar* | *("Sarcopenia" OR "muscle loss" OR "muscle wasting" OR "muscle atrophy" OR "skeletal muscle depletion" OR "cachexia" OR "frailty") AND ("Chronic Pancreatitis" OR "chronic pancreatitis" OR "pancreatic exocrine insufficiency" OR "PEI" OR "pancreatic disease") AND ("Body Composition" OR "body composition" OR "Skeletal Muscle" OR "skeletal muscle" OR "Exercise" OR "exercise" OR "handgrip strength" OR "grip strength" OR "gait speed" OR "appendicular lean mass" OR "ALM" OR "skeletal muscle index" OR "SMI" OR "DXA" OR "dual-energy X-ray absorptiometry" OR "bioelectrical impedance" OR "BIA" OR "CT" OR "MRI")* |
